# A left lateral body position increases pulmonary vein stress in healthy humans

**DOI:** 10.14814/phy2.15022

**Published:** 2021-09-23

**Authors:** Lisa A. Gottlieb, Dounia El Hamrani, Jérôme Naulin, Lorena Sanchez Y. Blanco, Jérôme Lamy, Nadjia Kachenoura, Bruno Quesson, Hubert Cochet, Ruben Coronel, Lukas RC Dekker

**Affiliations:** ^1^ IHU Liryc University of Bordeaux Pessac France; ^2^ AUMC Academic Medical Center Amsterdam the Netherlands; ^3^ Cardiology Department University Hospital Bordeaux, Pessac France; ^4^ Department of Radiology and Biomedical Imaging Yale University New Haven USA; ^5^ Laboratoire d’Imagerie Biomédicale Sorbonne Université CNRS INSERM LIB Paris France; ^6^ Department of Cardiovascular Imaging University Hospital Bordeaux, Pessac France; ^7^ University of Technology Eindhoven the Netherlands; ^8^ Cardiology Department Catharina Hospital Eindhoven the Netherlands

**Keywords:** body position, left lateral recumbence, myocardial stress, pulmonary veins

## Abstract

Pulmonary vein (PV) stretch is proarrhythmic for atrial fibrillation (AF). AF patients often report that a left lateral (LL) body position can trigger arrhythmia symptoms. Because the PV myocardium is thought to trigger AF, we hypothesized that the LL compared to the supine body position increases PV wall stress. Functional cardiac magnetic resonance imaging was performed in supine and LL recumbent body position in awake condition in healthy human volunteers (*n* = 20). Following a change from supine to LL position, the heart moved in an anterior‐LL direction in the thorax. The right superior PV diameter was increased by 19% (24.6 ± 3.1 vs. 20.7 ± 3.2 mm, *p* = 0.009) and left atrial (LA) volume was larger by 17% (61.7[15.4] vs. 51.0[17.8] ml, *p* = 0.015) in LL than supine position, respectively. The passive LA conduit fraction (normalized difference between maximum and pre‐contraction LA volume) increased by 25% in LL compared to supine position (19.6 ± 9.0 vs. 15.7 ± 7.6%, respectively, *p* = 0.016). Local wall stress in the PV regions increased in LL compared to supine position (overall mean: 1.01 ± 0.12 vs. 1.10 ± 0.10 arb. unit, LL vs. supine, position effect *p* = 0.041), whereas this was not the case in the LA walls (overall mean: 1.18 ± 0.31 vs. 1.21 ± 0.21 arb. unit, LL vs. supine, position effect *p* = 0.381). In conclusion, a left lateral body position increases PV myocardial stress during the atrial relaxation phase of healthy volunteers. These results have implications for the mechanisms of posture‐triggered AF.

## Graphical abstract

Atrial fibrillation can be triggered by taking a left lateral recumbent position. We show that a left lateral position causes anterior‐left localization of the heart and increases stress in the pulmonary veins.

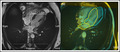

## 1 INTRODUCTION

Atrial fibrillation (AF) is a common cardiac arrhythmia with severe complications such as ischemic stroke and heart failure (Kannel and Benjamin, 2009). The population with AF is to increase to 18 million in Europe alone by 2050 (Rahman et al., 2014). Spontaneous ectopic activity is observed in the pulmonary veins (PV) in patients with paroxysmal AF (episode duration < 1 week) (Haissaguerre et al., 1998). Stretch of the PVs is known to increase the spontaneous activation rate in rabbits (Chang et al., 2007) and stretch of the atrial myocardium is proarrhythmic for AF by causing heterogeneous refractoriness and electrical conduction slowing (Ravelli and Allessie, 1997; Eijsbouts et al., 2003). More than 20% of symptomatic paroxysmal AF patients report that taking a specific body position triggers arrhythmia symptoms, and left lateral (LL) recumbence was a frequent AF‐triggering body position (Gottlieb et al., 2021). Because the heart is suspended in the mediastinum by the large blood vessel, including the PVs, we hypothesized that a change in recumbent body position from supine to LL position causes an increase in PV stress in humans.

We tested the hypothesis by obtaining functional cardiac magnetic resonance imaging (MRI) in supine and LL position in healthy volunteers that resembled an AF population in terms of age, sex, and body mass index (BMI) but had, however, no history of AF to avoid the interference of structural remodeling in the atria and to allow wall motion analysis comparisons in sinus rhythm.

Myocardial stress was evaluated by the maximal dimensions of the LA and PV in sinus rhythm (Laplace’s law, assuming unaltered intra‐cavity pressures in the two body positions). Also, we considered the localized passive deformation of the PV and LA myocardial walls during the passive atrial conduit phase (from mitral valve opening to immediately before atrial contraction) as an indicative of wall stress applied to the myocardium immediately before mitral valve opening.

## 2 MATERIALS AND METHODS

### 2.1 Volunteers

The study was ethically approved by the institutional national French review board (ref.2018/03, Comité de Protection des Personnes, Ile de France IV, France) and conformed to the declaration of Helsinki. Twenty random AF patient profiles including sex, age, and BMI were obtained from the University Hospital, Bordeaux, France. From a large group of volunteers (*n* = 81), we recruited volunteers each matching an AF‐profile thus having same sex, and similar age (2.6 ± 1.0 years difference), and BMI (0.6 ± 1.1 kg/m^2^ difference). The average age was 59.9 ± 1.9 years, 17 were male, and BMI was 26.5 ± 0.9 kg/m^2^. All volunteers gave written informed consent and reported being healthy.

### 2.2 MRI acquisition

MRI studies were conducted in supine and LL recumbent position on a 1.5Tesla system (MAGNETOM Aera, Siemens, Erlangen, Germany) with a 32‐channel body coil and an 18‐channel cardiac coil. First, the volunteer was placed in supine position. Cine imaging was performed by an ECG‐gated steady‐state free precession pulse sequence during breath‐hold in order to acquire a 4‐chamber stack (slice thickness 6 mm, no interslice gap) and a short‐axis stack (slice thickness 8 mm, no interslice gap) encompassing the entire heart. The following parameters were used: field of view = 300 × 230 mm^2^; acquisition matrix = 240 × 180; pixel size = 1.3 × 1.3 mm^2^; flip angle = 58°; bandwidth = 992 Hz/pixel; echo time = 1.34 ms; repetition time = 21.98 ms; GRAPPA acceleration factor of 3 with 75% partial k‐space acquisition. After the supine MRI study, the volunteer changed to a LL position on the scanner table, and the acquisition was repeated using the same scan parameters.

### 2.3 Image analysis

**FIGURE 1:**
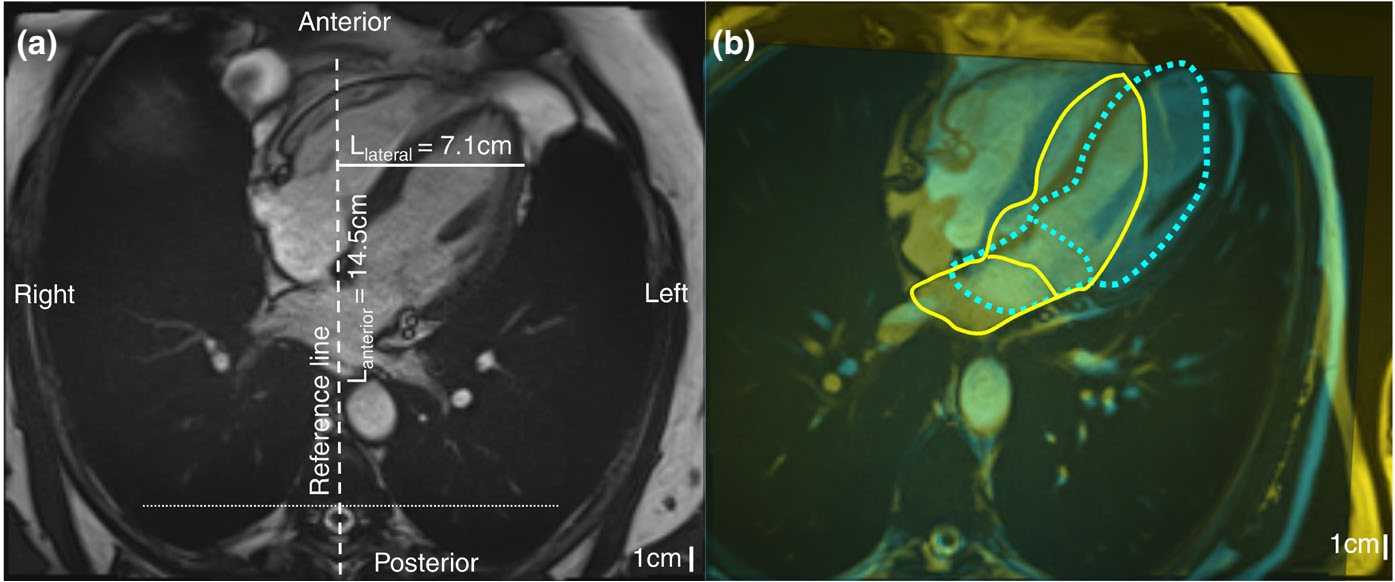
Heart position in the thorax. (a) A reference line going through the spinous process and the middle of the vertebral foramen was positioned in standard 4‐chamber images at the moment of end‐diastole. L_lateral_ was defined as the length of the normal to the reference line going through the most lateral part of the LV wall. L_anterior_ was defined as the length from the vertebral foramen to the intersection between the normal and the reference line. (b) Superimposition of standard 4‐chamber images in LL (blue) and supine position (yellow) shows that the heart shifts in an anterior‐LL direction in LL compared to in supine position. An endocardial contour in the LA and LV is drawn (yellow line: supine position; dotted blue: LL position)

The heart position in the thorax was investigated using Syngo.via software (Siemens, Erlangen, Germany) by positioning a reference line going through the spinous process and the middle of vertebral foramen in the standard 4‐chamber slice at the moment of ventricular end‐diastole (Figure 1). The reference line was orthogonal to a line going through the costal heads and the anterior border of the vertebral foramen. We constructed the normal to the reference line going through the most lateral part of the left ventricular (LV) wall. The length of this normal (L_lateral_) was considered the lateral position of the LV. The intersection between the normal and the reference line relative to the origin (vertebra) was the anterior position of the LV (L_anterior_).

**FIGURE 2:**
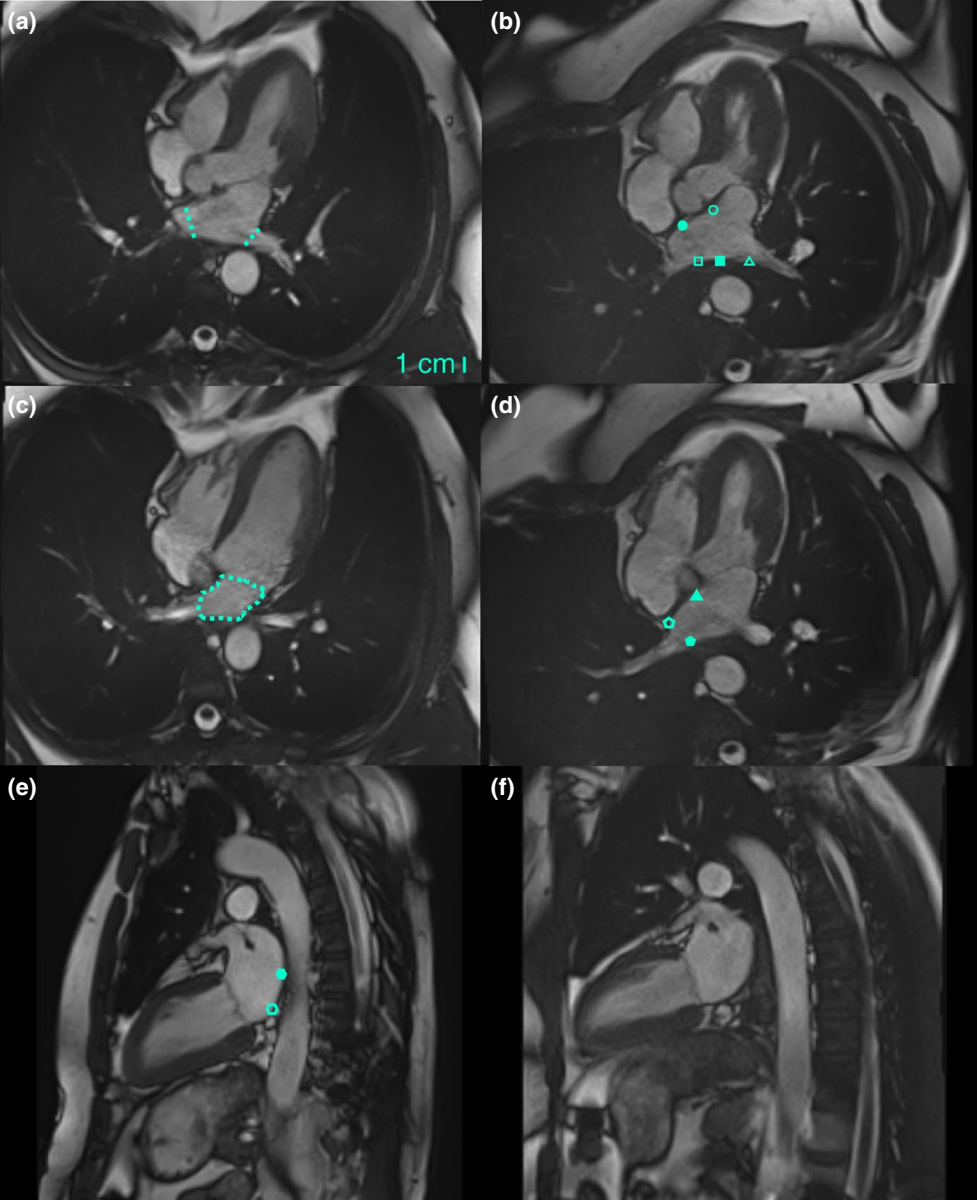
Representative 2‐ and 4‐chamber MRI in supine and LL position. (a) Four‐chamber MRI with RSPV and LSPV in supine position. Dotted lines indicate the PV diameters at the ostium. (b) Image in LL position with RSPV and LSPV. Regions for strain analysis were a LA septal (open circle), a RSPV anterior (closed circle), a RSPV posterior (open square), a LA posterior (closed square), and a LSPV posterior wall (open triangle). Note the sharp curvature of the LSPV anterior wall toward the LA appendage making tracking imprecise. (c) A 4‐chamber image with RIPV in supine position. Dotted line indicates the LA area used for volume estimation. (d) Image with RIPV in LL position. Regions for strain analysis were a LA septal (closed triangle), an RIPV anterior (open pentagon), and a RIPV posterior wall (closed pentagon). (e) Two‐chamber MRI in supine position. Strain regions were an inferior LA (open hexagon) and a posterior LA wall (closed hexagon). (f) Two‐chamber image in LL position. All images show the moment of maximum LA dilatation immediately before mitral valve opening. Lacking reproducibility of the MRI in the two body positions prevented us from analyzing all PVs

PVs remaining temporally in plane and with similar anatomical landmarks, such as the sternum, vertebrae, and aorta, in the two positions were included in the analysis. The PV diameter was measured at the ostium (Figure 2). The diameter of the right superior PV (RSPV) and the left superior SPV was analyzed twice by the first observer (LG) and once by a second observer (RC) in a blinded fashion. The intraobserver reproducibility of the PV diameter measurements was *r* = 0.705, H_0_ correlation: *p* < 0.001, mean bias = –1.2 mm, 95% limits of agreement from –8.5 to 6.2 mm, and the interobserver reproducibility was *r* = 0.486 , H_0_ correlation: *p* < 0.001, mean bias = 2.1 mm, 95% limits of agreement from –7.3 to 11.5 mm, thus showing an acceptable agreement. The repeated intra‐ and interobserver measurements generated similar data results.

Localized longitudinal strain (tangential deformation to the considered LA wall) throughout the cardiac cycle was analyzed with a feature‐tracking algorithm previously described after manual positioning of LA endocardial markers (> 30) on a single phase of the cardiac cycle (Evin et al., 2015, 2016). While excluding the PV ostia and LA appendage, we identified five wall regions in the PVs: an anterior and a posterior wall in RSPV, an anterior and posterior wall in right inferior PV (RIPV), and a posterior wall in LSPV. Also, five wall regions were defined in the LA: a posterior and an inferior LA wall in the 2‐chamber slice, a posterior and a superior septal LA wall in the 4‐chamber slice with RSPV, and an inferior septal LA wall in the 4‐chamber slice with RIPV (Figure 2). The beginning of the strain curve corresponded to the image immediately after atrial contraction, and we defined the passive conduit strain of the regional wall as the maximum magnitude in the 40–80% interval of the strain curve (from mitral valve opening to moment immediately before atrial contraction) (Williams et al., 2015). We considered the passive conduit strain as an indicative of wall stress applied to the myocardium immediately before mitral valve opening.

LA volumes were measured at maximum LA dilatation (maximal LA volume), immediately before atrial contraction (pre‐contraction LA volume), and immediately after atrial contraction (minimal LA volume) by tracing the endocardial LA contours on each slice of the 4‐chamber stack, while excluding the PVs and LA appendage (Figure 2). LA volumes were calculated as the sum of the resulting LA areas multiplied by the slice thickness.

The LA conduit fraction was defined as (maximal LA volume – pre‐contraction LA volume)/maximal LA volume × 100%, the LA active emptying fraction as (pre‐contraction LA volume – minimal LA volume)/pre‐contraction LA volume × 100%, the total LA ejection fraction as (maximum LA volume – minimum LA volume)/maximum LA volume × 100%, and the LA expansion index as (maximum LA volume – minimum LA volume)/minimum LA volume × 100%. Myocardial wall stress immediately before atrial contraction was evaluated by calculating Frank–Starling relations (LA stroke volume (pre‐contraction LA volume – minimal LA volume) as a function of pre‐contraction LA volume (preload) (Anwar et al., 2007).

LV volumes were measured on the short axis MRI stack by positioning end‐diastolic and end‐systolic endocardial LV contours from the apex to the slice below the mitral valve. Ejection fraction, stroke volume, and cardiac output were calculated.

### 2.4 Statistical analysis

Normality was tested by a Shapiro–Wilk test. Data are expressed as mean ± standard deviation or median [interquartile range] dependent on normality. Statistical testing of the cardiac volumes, mechanical functions, and heart rate was done with either a parametric two‐tailed paired Student’s *t*‐test or a nonparametric Wilcoxon signed‐rank test as appropriate.

A repeated measurements two‐way ANOVA was applied to the thoracic heart position parameters. Because not all PVs were identifiable and comparable in all volunteers, the PV diameters were tested with a mixed effect linear model, and Sidak’s method for multiple testing was applied. Local wall stress was calculated by logarithmic transformation of the maximal magnitude of the longitudinal strain curve during passive conduit in each region, and mixed effect linear models were used for statistical testing. Logarithmic regression was applied to Frank–Starling curves, and differences were statistically tested using an analysis of covariance.

Intra‐ and interobserver reproducibility was assessed by calculation of Pearson’s correlation coefficient (*r*) and by Bland–Altman analysis.

Statistically significant differences were considered with *p*‐values < 0.05. The data underlying this article will be shared on reasonable request to the corresponding author.

## 3 RESULTS

### 3.1 Anterior‐left heart position in the thorax

The MRIs of four volunteers were excluded from the analysis of the heart position in the thorax due to lack of visual vertebra in one of the two MRI acquisitions. Figure 1 shows an example of superimposition of standard 4‐chamber images of a volunteer in the LL and supine position. Note the anterior shift of the heart as well as the drop toward the LL thoracic wall. Following a change from the supine to LL position, L_anterior_ increased from 13.3 ± 2.0 to 14.6 ± 2.5 cm, respectively (*p* = 0.006) and L_lateral_ increased from 8.8 ± 0.9 to 9.9 ± 1.3 cm, respectively (*p* = 0.021) indicating that the LV took a more anterolateral position toward the LL thoracic wall in the LL position.

### 3.2 Larger RSPV in LL

**FIGURE 3:**
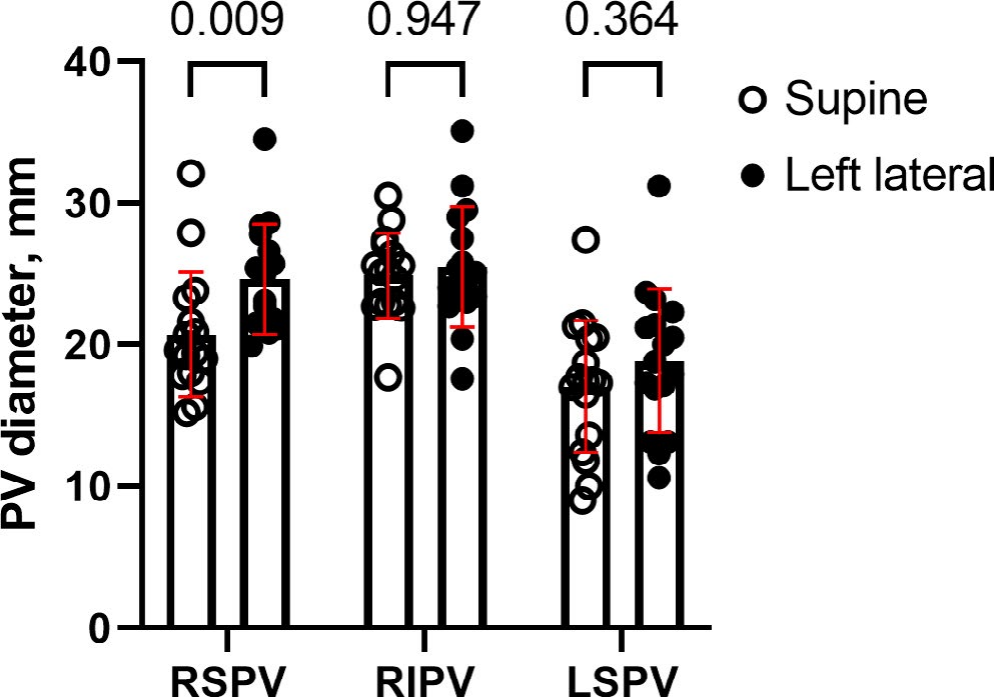
PV diameter in supine and LL position. The PV diameter was measured at the ostium at the moment of maximum LA dilatation immediate before mitral valve opening. The RSPV (*n* = 16) diameter increased in LL compared to supine position whereas the RIPV (*n* = 16) and LSPV (*n* = 17) diameter did not change. A mixed effect linear model was used for statistical testing. Sidak’s correction method for multiple testing was applied

We tested whether the anterior‐left localization of the heart altered the diameter of the PVs. Sixteen RIPVs, 16 RSPVs, 17 LSPVs, and 2 left inferior PVs (LIPV) were comparable in the 4‐chamber stacks. The low number of LIPVs did not permit statistical testing, and the LIPVs were thus omitted from the analysis. The maximum RSPV diameter increased from 20.7 ± 3.2 in supine position to 24.6 ± 3.1 mm in LL position (*p* = 0.009) whereas the LSPV and RIPV diameters were unaltered by the position change (Figure 3).

### 3.3 Enlarged left heart chambers

While the maximal LA volume was in mean 11ml larger (21% increase) in LL than in supine position, the LA pre‐contraction and minimal volumes were similar in LL and supine position (values in Table 1). Similarly, the end‐diastolic LV volume increased by a mean of 12 ml (9% increase) in LL compared to in supine position (Table 1). Stroke volume, LA and LV ejection fractions, and cardiac output were unchanged by position change (Table 1). Heart rate decreased significantly by a mean of 3bpm in LL compared to supine position (Table 1).

**TABLE 1:** Left heart chamber volumes and mechanics in supine and LL position

	Supine	LL	Supine vs. LL
Maximum LA volume, ml	51.0[17.8]	61.7[18.7]	*p* = 0.015
Pre‐contraction LA volume, ml	47.4 ± 13.9	49.9 ± 14.4	*p* = 0.242
Minimum LA volume, ml	28.5[17.3]	29.3[12.2]	*p* = 0.502
Total LA ejection fraction, %	48.5 ± 9.8	51.1 ± 10.8	*p* = 0.161
Active LA emptying fraction, %	39.2 ± 7.8	39.3 ± 9.8	*p* = 0.985
Passive LA conduit fraction, %	15.7 ± 7.6	19.6 ± 9.0	*p* = 0.016
LA expansion index, %	100.4 ± 36.3	113.2 ± 45.5	*p* = 0.119
LV end‐diastolic volume, ml	127.1 ± 27.0	139.0 ± 24.4	*p* = 0.009
LV end‐systolic volume, ml	48.2 ± 14.3	54.3 ± 18.5	*p* = 0.060
LV ejection fraction, %	62.0 ± 7.1	61.6 ± 8.2	*p* = 0.852
Stroke volume, ml	78.8 ± 18.9	84.7 ± 14.5	*p* = 0.085
Cardiac output, L	5.1 ± 1.5	5.2 ± 1.4	*p* = 0.739
Heart rate, bpm	64 ± 10	61 ± 9	*p* = 0.001

We evaluated LA and LV volumes and mechanics on functional cardiac MRI in healthy volunteers. Values are expressed as mean ± standard deviation or median [interquartile range] dependent on normality. Statistical testing was done with either a parametric two‐tailed paired Student’s *t*‐test or a nonparametric Wilcoxon signed‐rank test as appropriate. *N* = 20 in each parameter.

In summary, the enlargement in RSPV dimensions in LL compared to supine position coincided with augmented venous blood return thereby expanding the LA and LV during their respective relaxation phases.

### 3.4 Increased passive LA conduit fraction

**FIGURE 4:**
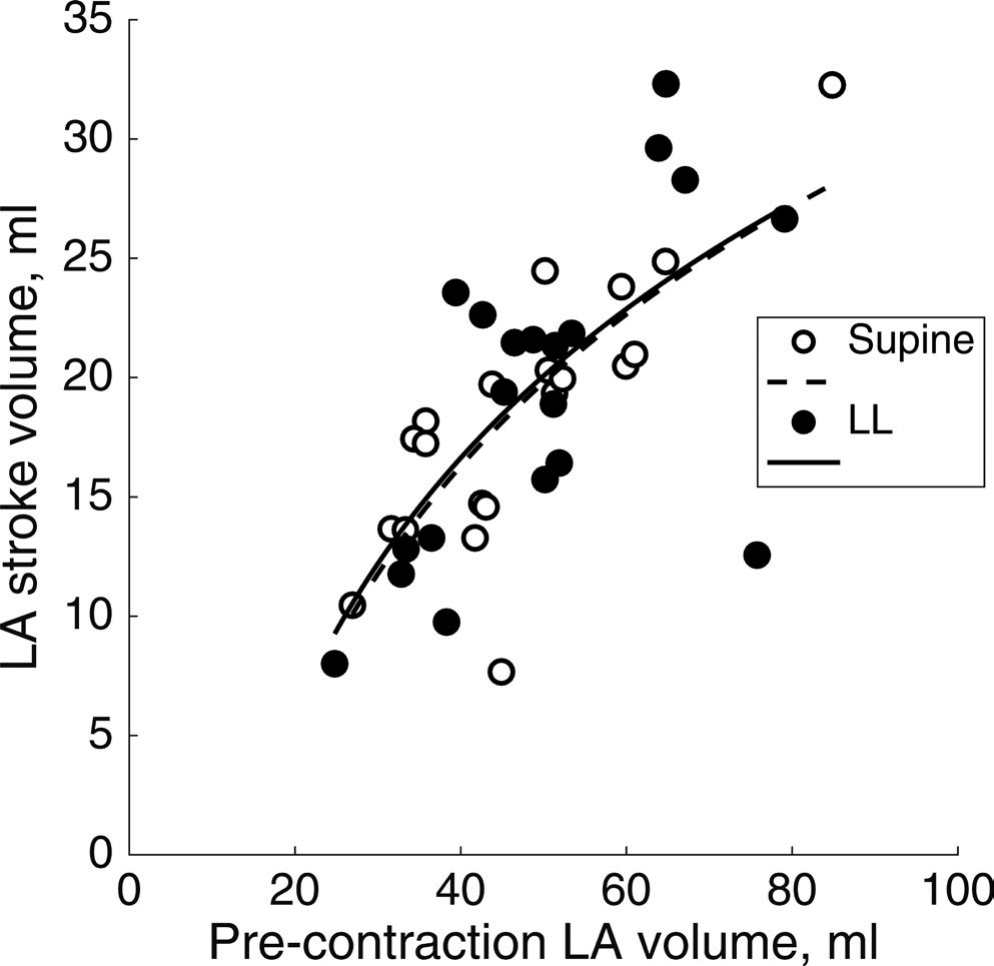
Frank–Starling relations. The global pre‐contraction wall stress in the LA evaluated by Frank–Starling relations did not change with a body position change from supine to LL position. Covariance analysis of the logarithmic regression curves showed no statistically significant differences (slope *p* = 0.954; intercept *p* = 0.942; *n* = 20 in each position)

The passive LA conduit fraction increased by 25% with a body position change from supine to LL position (Table 1). The active LA emptying fraction was similar in supine and LL position (Table 1). We investigated the Frank–Starling relations to evaluate pre‐contraction myocardial wall stress. The logarithmic regression curves of the Frank–Starling relation in supine and LL position did not differ (slope *p* = 0.954; intercept *p* = 0.942; Figure 4).

The increased passive LA conduit fraction in LL as compared to supine position indicated more wall stress at the moment of mitral valve opening. We, therefore, evaluated the local passive conduit strain of the PV and LA walls.

### 3.5 Increased local PV wall stress

**FIGURE 5:**
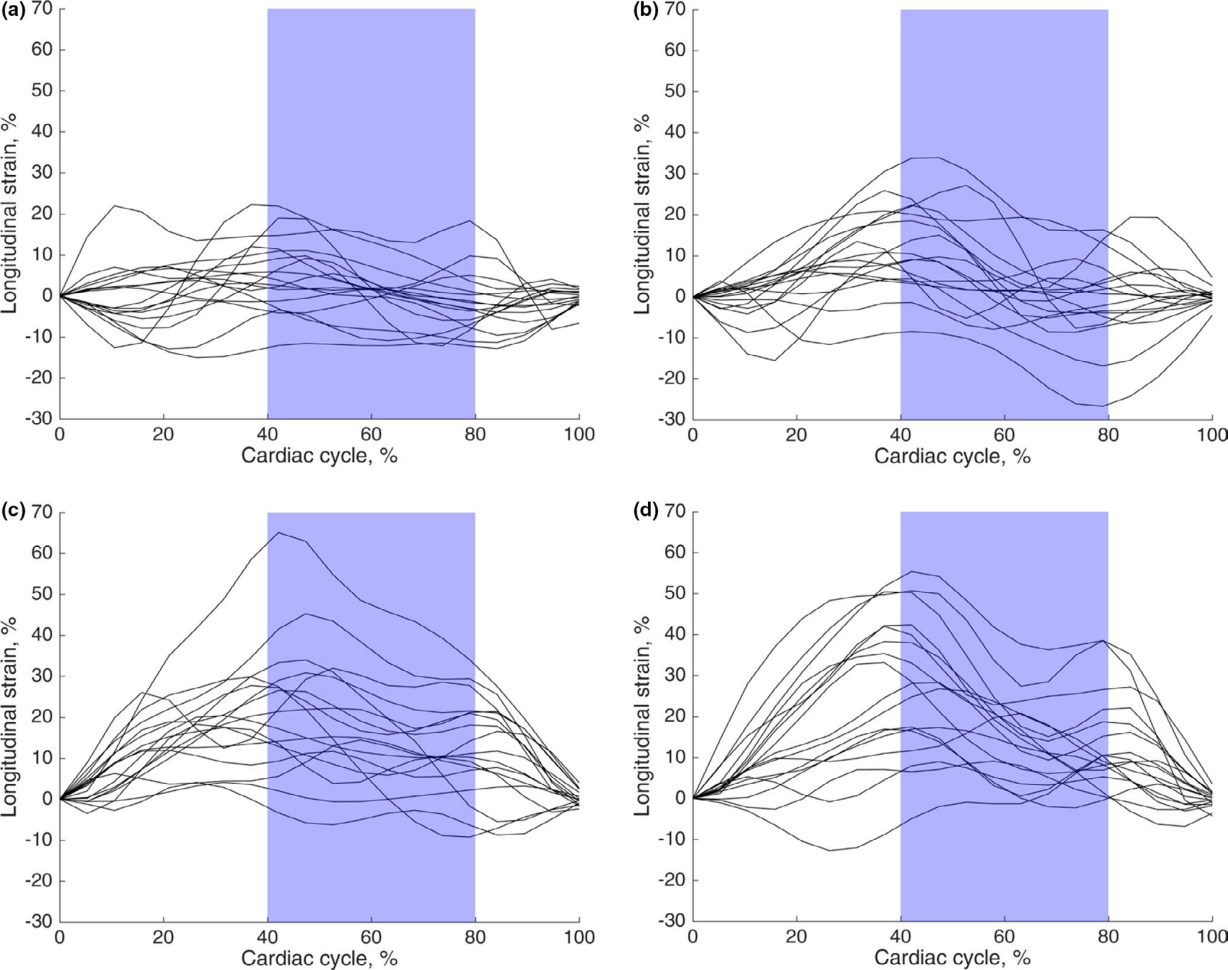
Localized longitudinal strain curves in PV and LA wall. (a) The longitudinal strain curves of the RIPV posterior walls in supine position. The blue shade reflects the 40–80% interval of the cardiac cycle that was considered the timing of the passive atrial conduit function. (b) The strain curves of the RIPV posterior walls in LL position. (c) The longitudinal strain curves of the LA septal wall (from the 4‐chamber slice with RIPV) in supine position. (d) The strain curves of the LA septal wall in LL position. *N* = 17 in each panel

Figure 5 depicts the longitudinal strain curves of the PV and the LA walls in supine and LL position. We considered the logarithmically transformed value of the maximum magnitude of localized longitudinal strain in the passive atrial conduit phase (40‐80% of cardiac cycle) as a marker of local wall stress (values in Table 2). Local wall stress in the PV regions increased in LL compared to supine position (overall mean: 1.01 ± 0.12 vs. 1.10 ± 0.10 arb. unit, LL vs. supine, position effect *p* = 0.041), whereas this was not the case in the LA walls (overall mean: 1.18 ± 0.31 vs. 1.21 ± 0.21 arb. unit, LL vs. supine, position effect *p* = 0.381).

**TABLE 2:** Local wall stress

Region	Wall	Supine	LL
PV^*^	RSPV anterior	1.08 ± 0.34	1.18 ± 0.25
	RSPV posterior	1.06 ± 0.42	1.02 ± 0.27
	RIPV anterior	1.13 ± 0.24	1.18 ± 0.24
	RIPV posterior	0.94 ± 0.31	1.15 ± 0.23
	LSPV posterior	0.82 ± 0.21	0.96 ± 0.26
LA^†^	LA posterior (4‐chamber)	0.74 ± 0.25	0.94 ± 0.24
	LA posterior (2‐chamber)	1.48 ± 0.33	1.22 ± 0.41
	LA inferior (2‐chamber)	1.49 ± 0.16	1.53 ± 0.20
	Superior LA septal	1.11 ± 0.26	1.14 ± 0.14
	Inferior LA septal	1.08 ± 0.26	1.26 ± 0.22

The logarithmically transformed values of maximum deformation during the passive atrial conduit phase was considered an indicative of local wall stress. Values are expressed as mean ± standard deviation and are in arbitrary unit. A mixed effect linear model showed a statistically significant effect of body position on wall stress of the PV regions (^*^body position effect *p* = 0.041), while this was not the case in the LA regions (^†^body position effect *p* = 0.381). The number of data points in each wall region was: RSPV slice *n* = 15; LSPV slice *n* = 16; RIPV slice *n* = 17; Two‐chamber *n* = 14.

## 4 DISCUSSION

We observed that a LL recumbent position led to a more anterior‐LL localization of the heart in the thorax. We reasoned that because the heart is suspended by the large arterial and venous vessels in the mediastinum, such an altered heart localization in the LL body position alters myocardial stress locally in the PVs. Indeed, a change from supine to LL position led to enlargement of the RSPV and LA during atrial relaxation. The passive LA conduit fraction increased in LL position and was associated with more passive conduit deformation in the walls of the PVs, but not of the LA, thereby reflecting body position‐dependent disparate changes in localized myocardial wall stress.

### 4.1 Atrial stress and body position

Others similarly have reported that LL position causes an increase in LA dimensions as compared to supine position in younger humans (Khan et al., 2017, Pump et al., 2002, Wieslander et al., 2019). Also, the left PVs are observed to be larger in LL than in right lateral position, and vice versa (Wieslander et al., 2019). Ohta et al. (2002) have described that the systolic blood flow in the left PVs in AF patients, while in AF, is increased as compared to the right PVs in LL position, whereas this difference is not observed in supine position. Thus, LA and PV dimensions and flow depend on body position.

Laplace’s law states that an increase in dimensions augments wall stress under the assumption of unchanged pressure. We consider that the intra‐atrial pressure did not alter with the body position change because our subjects reported being healthy and without mitral valve disease and pulmonary hypertension. Therefore, the larger maximum dimensions of the LA and RSPVs occurring during atrial relaxation in LL position implies that the wall stress was higher.

Atrial conduit function is the passive blood transport from the atrium to the ventricle during early ventricular diastole and is influenced by compliance of the atrial tissue (Hoit, 2014). Tissue recoil (i.e., inversely related to compliance) depends on the pressure and volume, and thereby wall stress. We report that the PV myocardial walls passively deformed more in LL than supine position upon pressure release by mitral valve opening thereby emphasizing a higher PV wall stress in LL position. However, the wall stress immediately before atrial contraction was similar in supine and LL as reflected by similar Frank–Starling relations and active LA emptying fractions in the two body positions.

### 4.2 Electrophysiological consequences of myocardial stress

Chang et al. (2007) report that stretching of the PVs increases the spontaneous activation rate. Others describe that the highest frequency of AF occurred in the PVs during an atrial volume dilatation (Kalifa et al., 2003). Atrial stress by volume expansion also increases heterogeneity in refractoriness and in electrical conduction in the atrial tissue (Ravelli and Allessie, 1997, Eijsbouts et al., 2003). The dilatation‐induced heterogeneity in conduction can be a result of heterogeneous distribution of wall stress because thinner regions of the atria stretches more than thicker regions during volume expansion (Satoh and Zipes, 1996). Unfortunately, measurement of localized differences in atrial myocardial thickness (radial strain) was not feasible in our MRI data because the in‐plane resolution was 1.3 × 1.3 mm^2^. Still, we observed that a LL body position caused longitudinal strain changes in the PV, but not in the LA walls. Such disparate changes in deformation of the PVs and LA walls can enhance a proarrhythmic setting by augmenting electrical heterogeneity.

### 4.3 Posture‐triggered AF

Symptomatic and asymptomatic AF episodes occur often during rest (Rosso et al., 2010). Increase in parasympathetic nervous activity has been proposed to be the trigger of nocturnal AF (Rosso et al., 2010). However, the individual’s body position during rest may also play a role in AF triggering. Indeed, 22% of symptomatic paroxysmal AF patients report that arrhythmia symptoms are specifically triggered by taking a specific body position, and the LL recumbent position was frequent (Gottlieb et al., 2021).

AF itself initiates structural atrial remodeling leading to atrial dilatation, loss of contractile fibers, and fibrotic tissue formation (Ausma et al., 1997, Boldt et al., 2004). This is reflected in the observation that AF patients have larger PVs and LA than healthy controls (Tsao et al., 2001, Gupta et al., 2014). Also, collagen fibers intersperse the cardiomyocytes in the PVs of AF patients (Hassink et al., 2003), and collagenous tissue is stiffer than myocardium (Connelly et al., 1991). Therefore, a LL position may cause even higher increases in myocardial stress in compliant (non‐fibrotic) regions in diseased atria in AF patients. Indeed, the border zone between fibrotic infarct and vital myocardium deforms more than the infarct area itself (Bertini et al., 2010). In patients with AF, atrial wall stress is estimated to peak in the PVs (Hunter et al., 2012). Moreover, the increased deformation of the PV walls occurred during the passive atrial conduit phase which corresponds to the timing of recovery from refractoriness that is a particularly vulnerable moment for arrhythmia induction by afterdepolarizations (Schotten et al., 2011). Thus, the observed positional changes in PV diameter are likely an underestimation of what happens in patients with AF. Our positional study therefore needs to be repeated in patients with paroxysmal AF. We intentionally recruited healthy volunteers to study the causal relation between body position and atrial stress without the interference of structural AF remodeling and without the concomitant decrease of statistical power.

Posture‐triggered atrial arrhythmias may also depend on alterations in autonomic nervous activity. The heart rate of the volunteers was slightly lower in LL than in supine position, although these changes were smaller than during a stand‐up test (Hofsten et al., 1999). This has previously been described in an elderly population (Sasaki et al., 2017). Mechanical preload obstruction in supine position could initiate an autonomic nervous adjustment of the cardiac output by increase in heart rate. However, the heart rate variability, as a marker of autonomic nervous activity, is unaltered in LL position compared with supine (Sasaki et al., 2017), thus excluding a major change in autonomic tone.

## 5 CONCLUSION

When the subject changed from a supine to a LL position, the heart moved in an anterior‐LL direction in the thorax causing an enlargement in RSPV dimensions. This was associated with a larger passive PV wall deformation during atrial relaxation leading to an increase in the passive LA conduit fraction in LL position. Therefore, LL position increases PV wall stress. These changes potentially contribute to a proarrhythmic electrophysiological effect in LL position.

## CONFLICTS OF INTERESTS

The authors have declared no conflicts of interest.
